# Supplementary material for: Mechanism of ASF1 engagement by CDAN1
Source: Nat Commun. 2025 Mar 16;16:2599. doi: 10.1038/s41467-025-57950-z (PMC11911400; doi:10.1038/s41467-025-57950-z)
Supplement: Supplementary file 1 — Supplementary Information [file 41467_2025_57950_MOESM1_ESM.pdf]

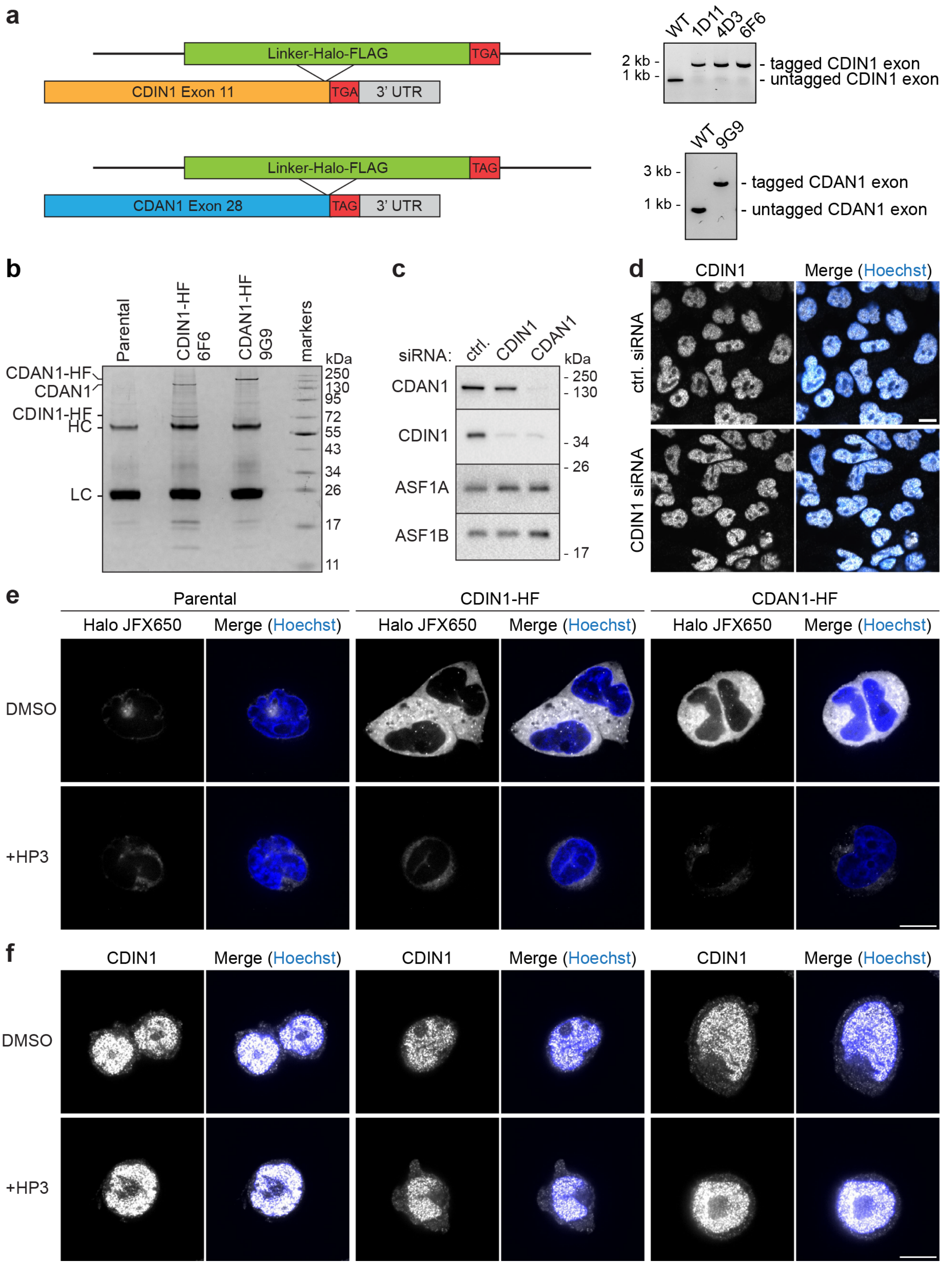

Supplementary Figure 1. Characterization of endogenous CDAN1 and CDIN1. a, Scheme (left) for endogenously tagging CDIN1 and CDAN1 with a C-terminal HaloTag-FLAG (HF) tag and PCR validation of knock-in clones (right). **b,** SDS-PAGE and Coomassie staining of anti-FLAG resins after immunoprecipitations of lysates from the indicated parental, CDIN1-HF, and

CDAN1-HF Flp-In 293 T-REx cells. HC – heavy antibody chain, LC – light antibody chain. Note: CDIN1-HF copurifies an approximately equimolar amount of endogenous CDAN1; endogenous CDIN1 between LC and HC. **c,** SDS-PAGE and immunoblotting of lysates of Flp-In 293 T-REx cells treated with control (ctrl.) siRNAs or siRNAs against CDIN1 or CDAN1. Note: knocking down CDAN1 destabilizes CDIN1, but not vice versa; representative of 3 independent replicates. **d,** Immunofluorescence using anti-CDIN1 antibody (gray) of Flp-In 293 T-REx cells treated with ctrl. siRNA (siNeg) or siRNA against CDIN1, co-stained with Hoechst (blue). Note: the nuclear signal observed with the anti-CDIN1 antibody does not decrease after knocking down CDIN1. **e,** Live-cell imaging of the indicated cells labeled with the JFX650 HaloTag ligand (gray) and Hoechst (blue) after treatment with DMSO or 500 nM HaloPROTAC3 (HP3) for 48 hr. Note: the specific cytosolic HaloTag signal in CDIN1-HF and CDAN1-HF cells is abolished after HP3 treatment. **f,** Immunofluorescence of cells from **e** after fixation using anti-CDIN1 antibody (gray) and Hoechst (blue). Note: signal from the antibody is observed in all conditions and does not decrease in any cell line after HP3 treatment. Scale bar, 10 μm for all images.

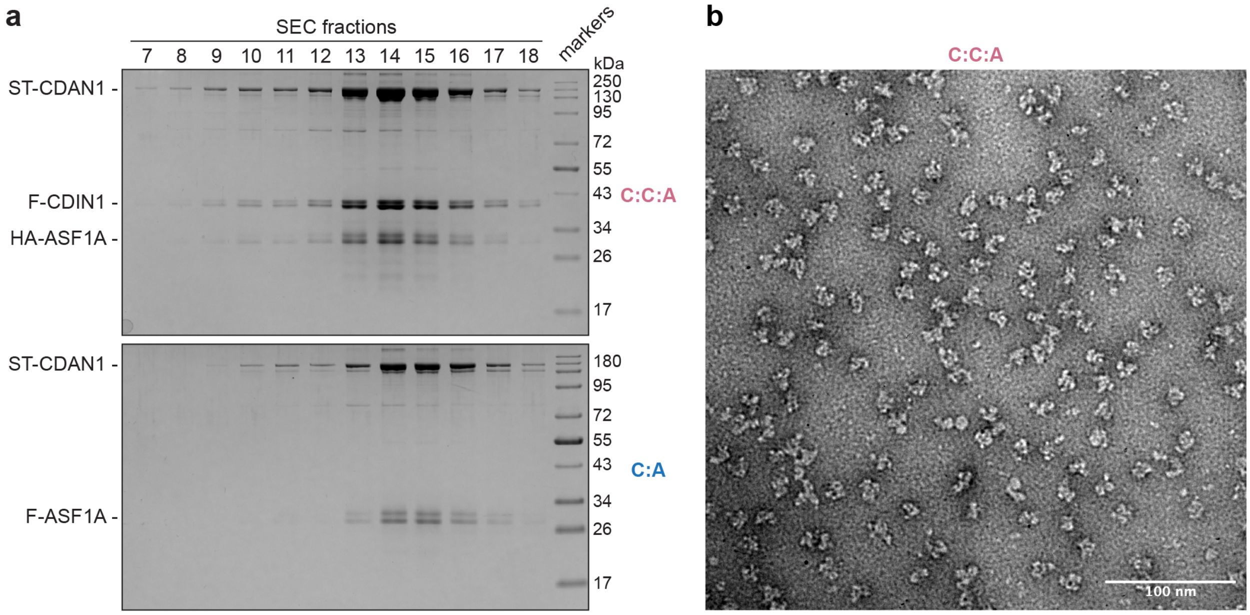

c

Complex	SEC-MALS molar mass	SEC-MALS molar mass range	theoretical molar mass C:(C:)A stoichiometry			polydispersity
			**2:(2:)2**	**2:(2:)3**	**2:(2:)4**	
C:A	364.3 ± 0.40%	362.8 - 365.8	331.7	**357.7**	383.7	1 ± 0.50%
C:C:A	432.1 ± 0.40%	430.4 - 433.8	404.4	**431.4**	458.4	1 ± 0.53%

Supplementary Figure 2. Characterization of purified CDAN1 complexes. a, Size exclusion chromatography fractions of purified CDAN1-CDIN1-ASF1A (C:C:A; top) or CDAN1-ASF1A (C:A; bottom) analyzed by SDS-PAGE and Coomassie staining show comigration of the complex components. **b,** Representative negative stain EM image of the C:C:A complex. **c,** Table showing size exclusion chromatography with multi-angle light scattering (SEC-MALS) measurements of the molar mass (kDa) of the C:A or C:C:A complex, together with the theoretical molar mass of each complex with the indicated stoichiometries.

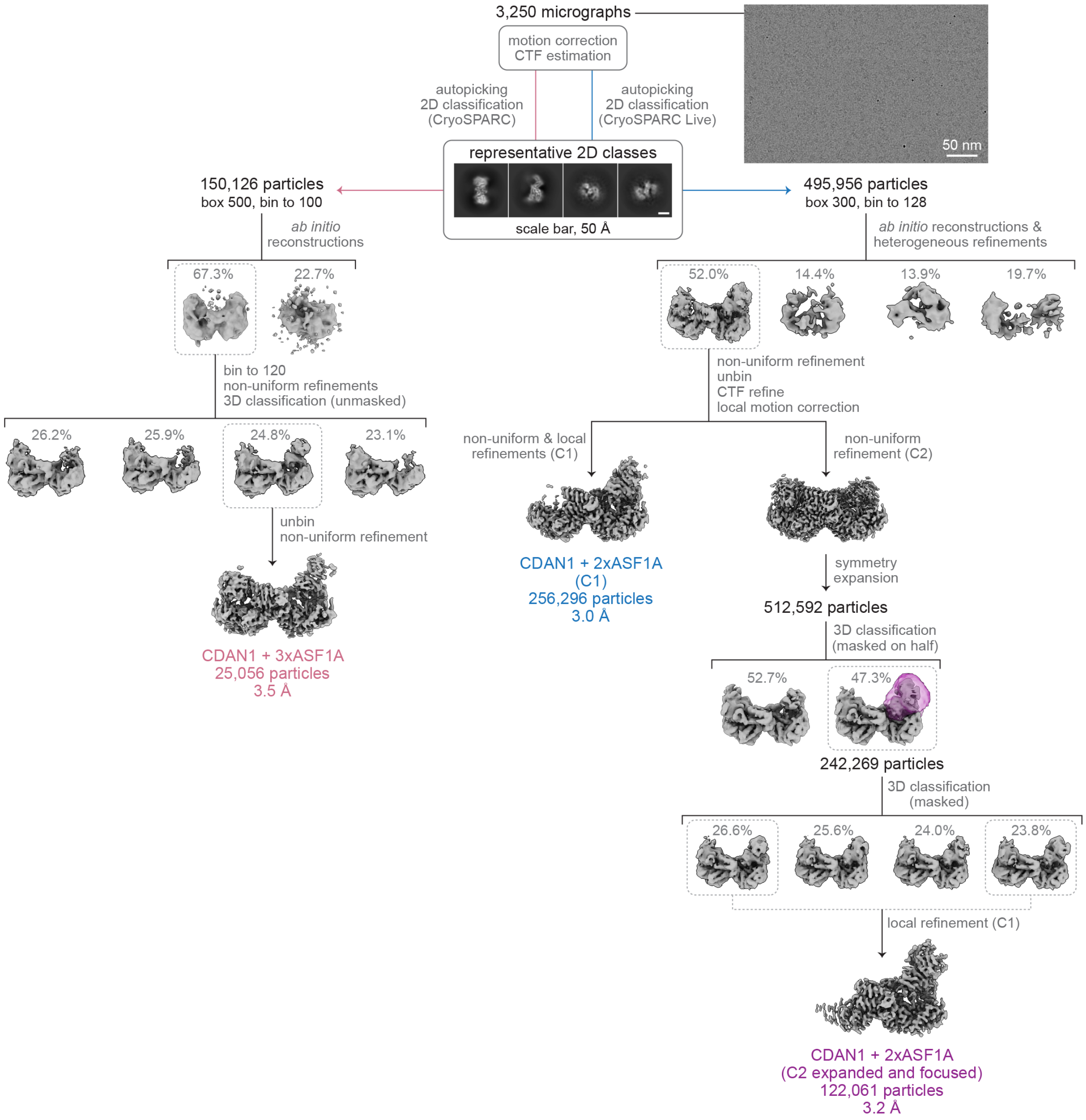

Supplementary Figure 3. Cryo-EM data processing summary. Overview of single-particle cryo-EM data processing and classification pipeline. The map with three ASF1A molecules was obtained from a separate autopicking and 2D classification pipeline using a larger box size (left) than the other maps, which were obtained from initial processing in CryoSPARC Live. Because asymmetry was consistently observed in the maps, the refinement with C2 symmetry was only used for symmetry expansion. The expanded particle set was then subjected to 3D classification masked on elements on one half of the map to obtain higher resolution insights into how two ASF1A molecules are stacked on one side of the CDAN1 dimer.

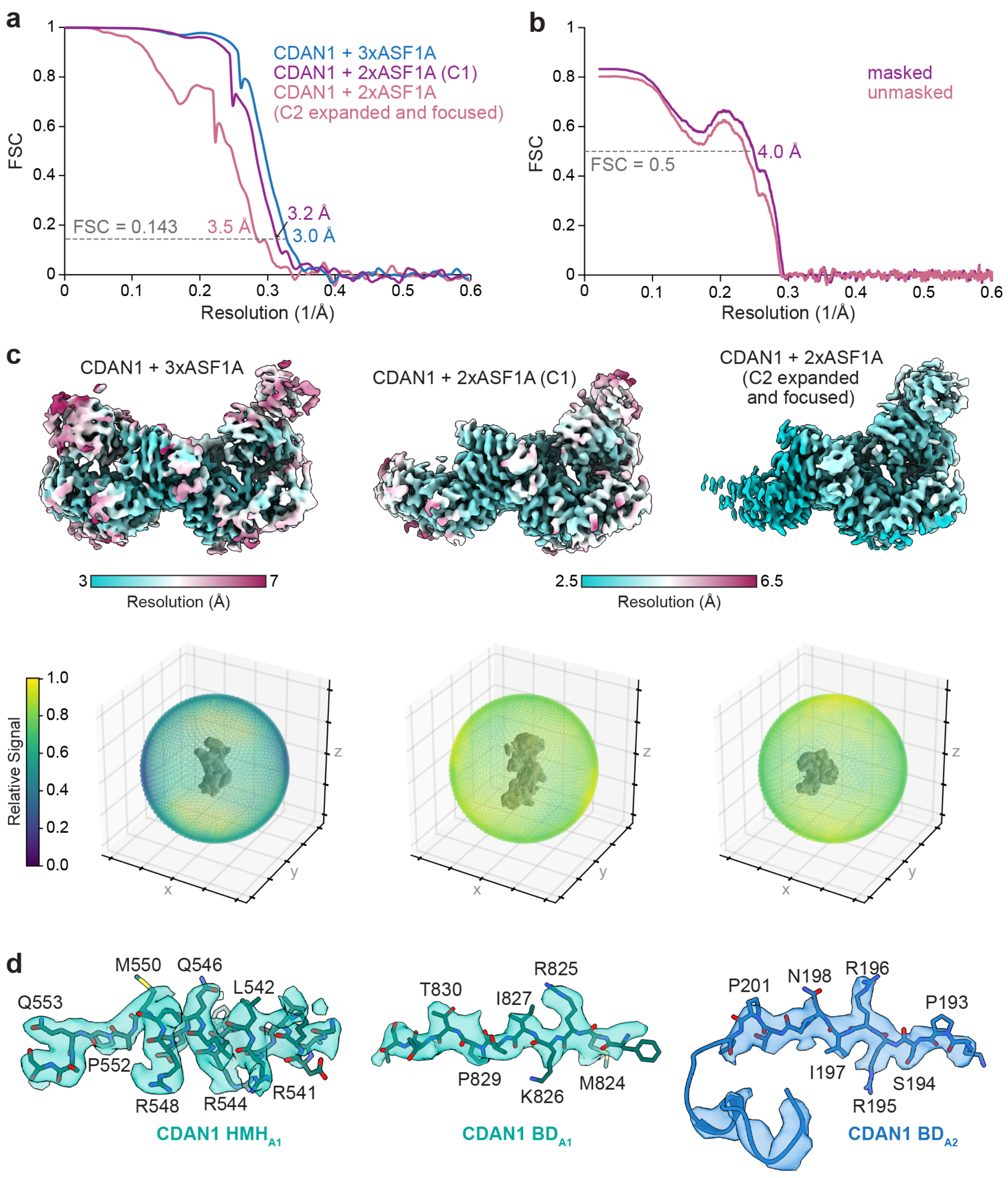

Supplementary Figure 4. Quality of maps and models. a, Fourier shell correlation (FSC) vs. resolution (1/Å) curves for the indicated cryo-EM maps. **b,** Model vs. map FSC curves. **c,** The indicated cryo-EM maps colored by local resolution (top) and shown with the angular distribution of particles used in the final reconstructions (bottom). **d,** Model to map fits for the indicated CDAN1 elements in MIF4G2 (teal) or MIF4G1 (blue).

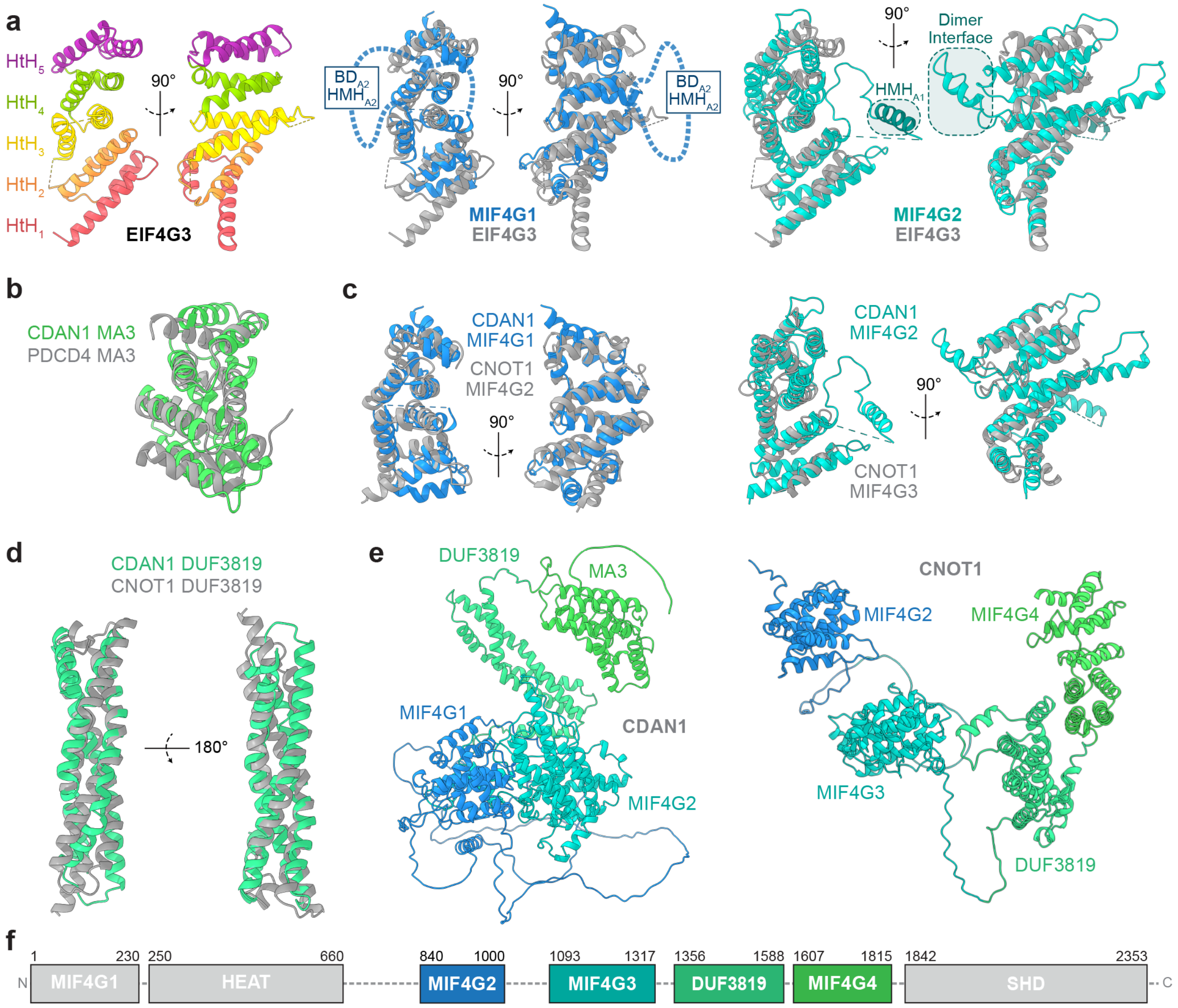

Supplementary Figure 5. CDAN1 domain analysis. a, Canonical MIF4G domain of eIF4G3 (PDB 1HU3) with each helix-turn-helix (HtH) motif colored separately (left) or superposed (gray) with CDAN1 MIF4G1 (middle; blue) or CDAN1 MIF4G2 (right; teal). **b,** Canonical MA3 domain of PDCD4 (PDB 2RG8, gray) superposed with the CDAN1 MA3 domain (green; Dali Z-score 7.8). **c,** Superposition of CDAN1 MIF4G1 (blue) with CNOT1 MIF4G2 (gray, left) or of CDAN1 MIF4G2 (teal) with CNOT1 MIF4G3 (gray, right). **d,** Superposition of the three-coil bundle domain of unknown function (DUF3819) predicted to be present in both CDAN1 (sea green) and CNOT1 (gray). **e,** Alphafold2 model of CDAN1 (left) or the central region of CNOT1 (right) colored by domain: CDAN1 MIF4G1 or CNOT1 MIF4G2 (blue), CDAN1 MIF4G2 or CNOT1 MIF4G3 (teal), DUF3819 (sea green), and CDAN1 MA3 or CNOT1 MIF4G4 (green). **f,** Domain scheme of CNOT1 with the central region similar to CDAN1 colored as in **e**.

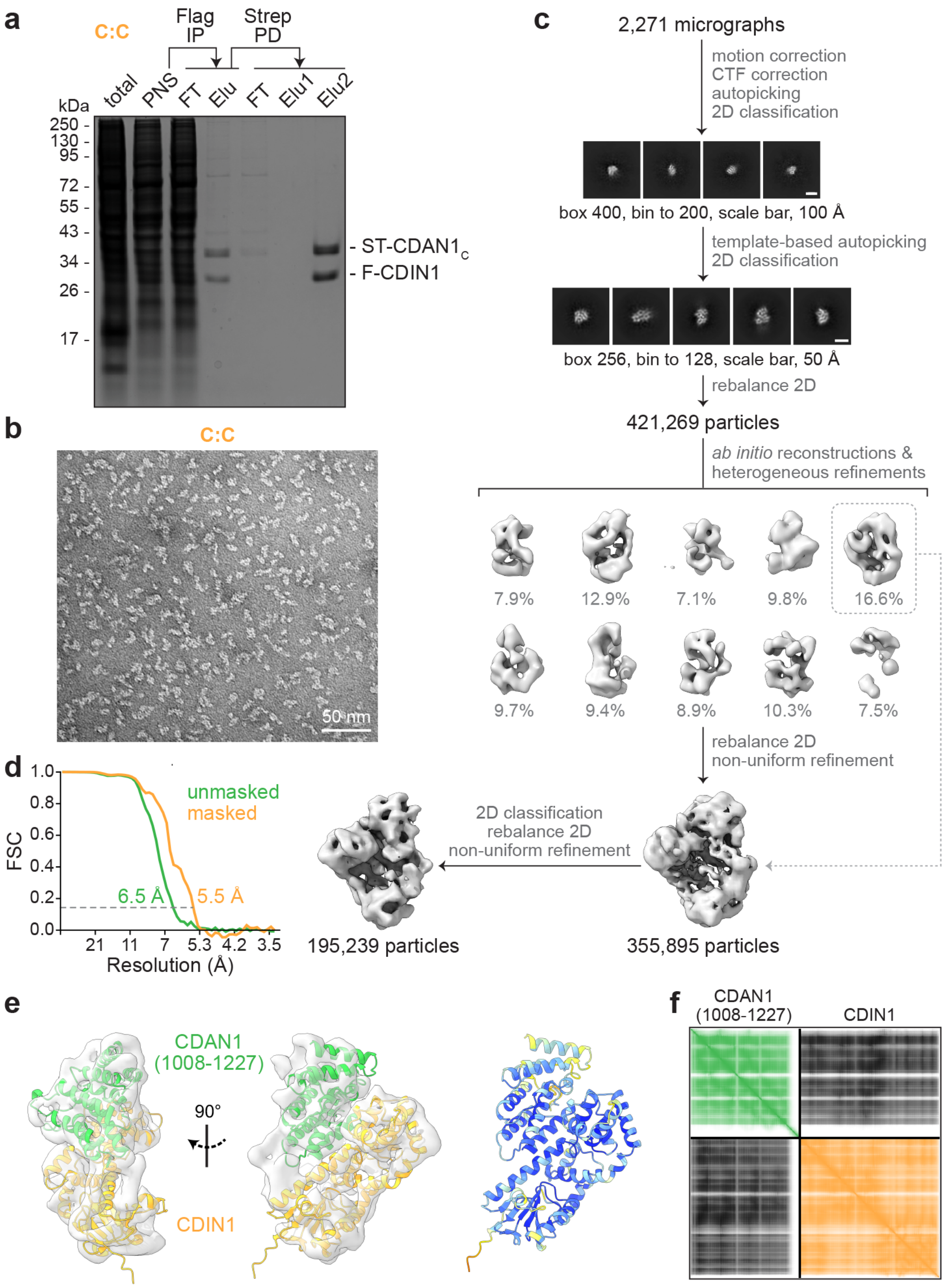

Supplementary Figure 6. Analysis of the CDAN1-CDIN1 interaction interface. a, The Strep-tagged MA3 domain of CDAN1 (ST-CDAN1$_C$;, residues 1008-1227) was co-expressed with FLAG-tagged CDIN1 (F-CDIN1) in Expi293 cells by transient transfection. The cells were lysed, and the post-nuclear supernatants (PNS) were subjected to anti-FLAG immunoprecipitation (FLAG IP) followed by Strep-Tactin pulldowns (Strep PD) to purify the CDAN1$_C$-CDIN1 (C:C) complex. The total lysate, PNS, flow-through (FT), and elution (Elu) samples were analyzed by SDS-PAGE and Coomassie staining. **b,** Representative negative stain EM image of the C:C complex. **c,** Summary of cryo-EM data processing scheme for the C:C complex. **d,** Fourier shell correlation (FSC) vs. resolution (Å) curves for the cryo-EM map of the C:C complex. **e,**

Alphafold3 model of the C:C complex colored by chain (CDAN1 – green, CDIN1 – light orange; left) or pLDDT values (right) docked into the cryo-EM map (left, transparent gray)**. f,** Predicted alignment error (PAE) plot of the Alphafold3 prediction of the C:C complex colored as in e.

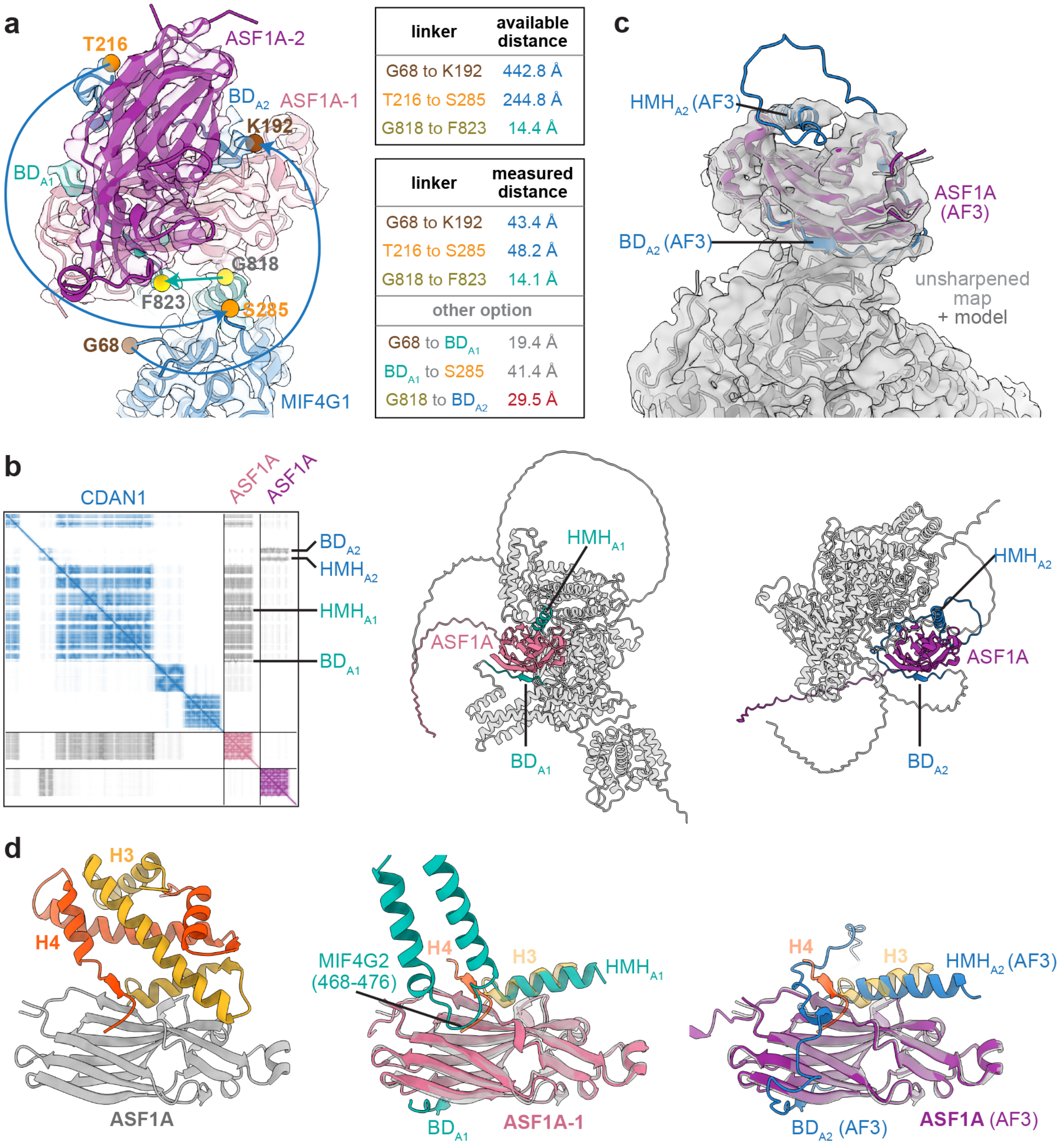

linker	available distance
G68 to K192	442.8 Å
T216 to S285	244.8 Å
G818 to F823	14.4 Å

linker	measured distance
G68 to K192	43.4 Å
T216 to S285	48.2 Å
G818 to F823	14.1 Å
other option	
G68 to BD$_{A1}$	19.4 Å
BD$_{A1}$ to S285	41.4 Å
G818 to BD$_{A2}$	29.5 Å

Supplementary Figure 7. CDAN1 interactions with ASF1A. a, Overlay of the structural model of CDAN1 bound to two ASF1A with the focused cryo-EM map contoured at 4.4σ, viewed from the top of the ASF1A stack (left). A >200 amino acid loop (residues 64-298) extending from CDAN1 MIF4G1 (blue), which harbors the previously identified B-domain sequence (BD$_{A2}$) with a conserved extension (collectively, residues 192-216 are modeled) and a putative H3 mimic helix (see **c**), is sufficiently long to engage the distal ASF1A-2 (purple). In contrast, the BD$_{A1}$ sequence (residues 823-832, teal) can only reach ASF1A-1 (pink) from the modeled structured domain ending at residue 818. Arrows indicate the direction of unmodeled linker regions between the indicated residues. The theoretical distances that unmodeled linkers on either side of the B-domain sequences can span (assuming 3.6 Å/amino acid; top) and the measured direct distances between the indicated residues or elements (bottom) are listed on the right. **b,** Left: Predicted alignment error (PAE) plot (left) of the Alphafold3 (AF3) prediction of one CDAN1 (blue) with two ASF1A molecules (pink and purple). Right: Images show each ASF1A molecule

colored as in Fig. 3 with predicted interactions with HMH_{A1} and BD_{A1} (teal; middle) or HMH_{A2} and BD_{A2} (blue; right). Each view is aligned on the respective ASF1A. **c,** The Alphafold3 (AF3) prediction of ASF1A (purple) bound to the putative HMH_{A2} (residues 247-260; blue) of CDAN1 superposed to ASF1A-2 of our structural model (dark gray) and docked into the unsharpened CDAN1 + 3xASF1A map contoured at 6.8σ (transparent gray). Note: unmodeled density corresponding to the position of HMH_{A2} predicted by AF3. **d,** Structural model of ASF1A (gray) bound to a histone H3 (gold)-H4 (dark orange) dimer (PDB 2IO5; left) superposed (transparent) with our structural model of ASF1A-1 (pink) and binding elements of CDAN1 (teal; middle) or the Alphafold3 prediction of ASF1A (purple) bound to the extended BD_{A2} and HMH_{A2} (blue; right). Note: potential clash of the H4 tail with a CDAN1 MIFG2 loop (residues 468-476; middle) on ASF1A-1 and with the predicted extension of BD_{A2} (right).

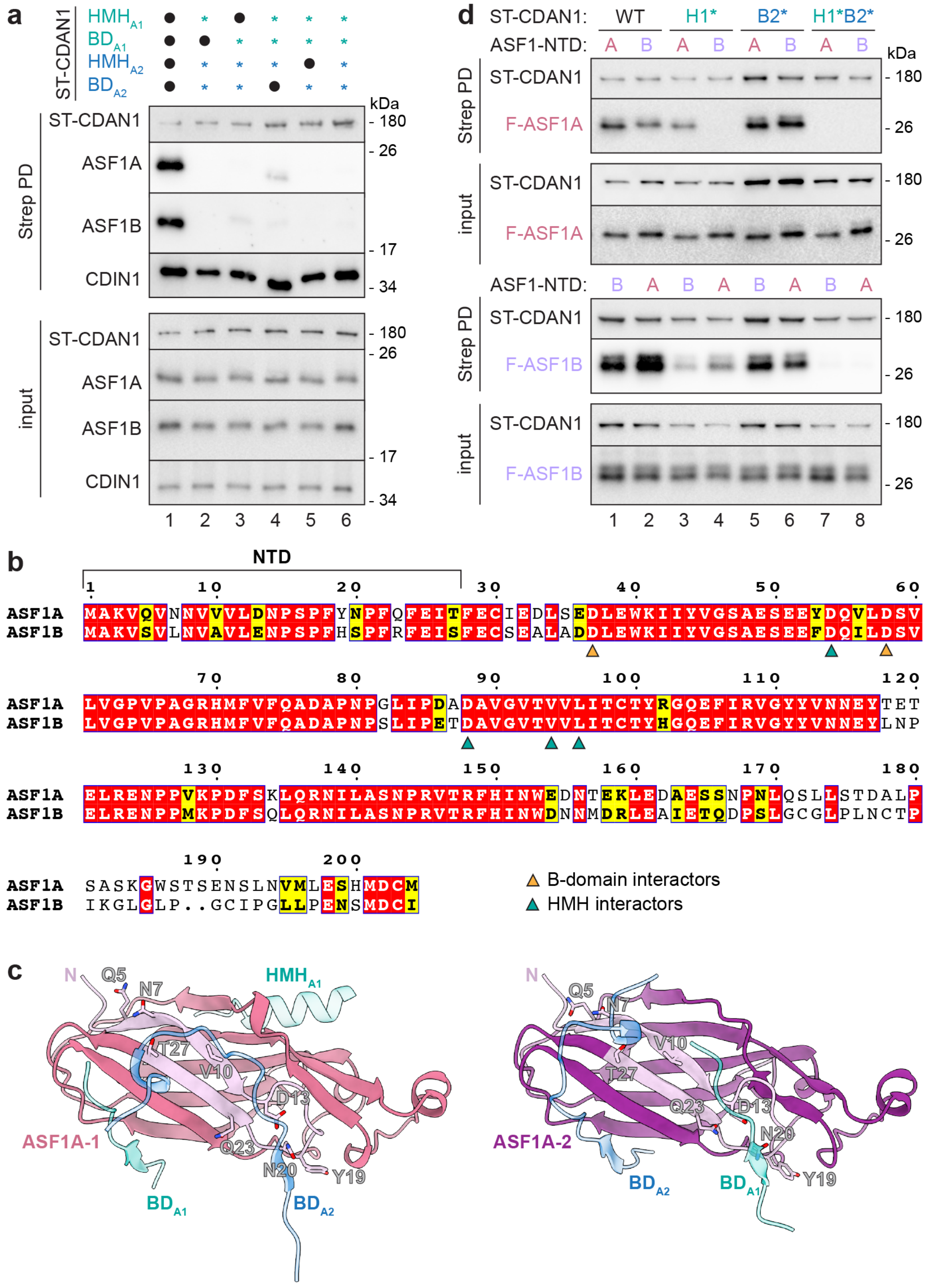

Supplementary Figure 8. ASF1A and ASF1B comparisons. a, Flp-In 293 T-REx cells transiently transfected with Strep-tagged CDAN1 (ST-CDAN1) variants without (filled circles) or with (*) mutations in the indicated CDAN1 domains (see Fig. 5a) were lysed (input) and subjected to Strep-Tactin pulldowns (PD) before analysis by SDS-PAGE and immunoblotting; representative of 3 independent replicates. Note: No CDAN1 variants with mutations in any combination of three or more of the binding elements pull down ASF1A or ASF1B. **b,** Sequence

alignment of ASF1A and ASF1B. The N-terminal domain (NTD; the first 27 amino acids) and residues that mediate interaction with B-domains (orange triangle) or with histone H3 or H3 mimic helices (HMH; teal triangles) are indicated. Identical residues are highlighted in red; similar residues are highlighted in yellow[66]. **c,** Placement of the CDAN1 B-domains (BD_{A1}, teal, and BD_{A2}, blue) and HMH_{A1} relative to ASF1A-1 (pink; left) and ASF1A-2 (purple; right). The NTD (lavender) of each ASF1A is indicated. Residues that differ from ASF1B are in stick representation and labeled. **d,** Wildtype (WT) ST-CDAN1 or ST-CDAN1 variants containing mutations in HMH_{A1} (H1*) and/or BD_{A2} (B2*; see Fig. 5) were co-transfected with FLAG-tagged ASF1A (F-ASF1A; top) or ASF1B (F-ASF1B; bottom) variants with the indicated NTD. Cell lysates before (input) or after Strep PD were analyzed by SDS-PAGE and immunoblotting; representative of 3 independent replicates.

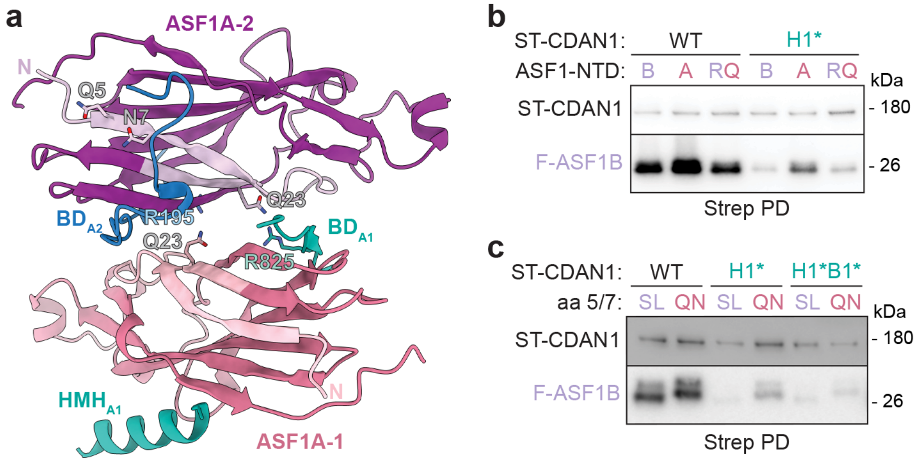

Supplementary Figure 9. Potential ASF1 NTD interactions. a, Structural model showing the stacked configuration of ASF1A-1 (pink) and ASF1A-2 (purple) with the indicated CDAN1 binding elements (teal and blue). The N-terminal domain (NTD) of ASF1A-1 and ASF1A-2 are light pink and lavender, respectively. Q23 on each ASF1A, Q5 and N7 on ASF1A-2, and R195 and R825 in the B-domains of CDAN1 are shown in stick representation and labeled. **b,** Wildtype (WT) ST-CDAN1 or ST-CDAN1 containing mutations in HMH_{A1} (H1*) were co-transfected with FLAG-tagged ASF1B (F-ASF1B) variants containing the ASF1B NTD (B), ASF1A NTD (A), or an R23Q point mutation (RQ). Strep pulldowns (PD) from cell lysates were analyzed by SDS-PAGE and immunoblotting. Note: the ASF1A NTD swap but not the R23Q mutation enhances association with H1* ST-CDAN1; representative of 3 independent replicates. **c**, As in **b** but with WT, H1*, and ST-CDAN1 containing mutations in both HMH_{A1} and BD_{A1} (H1*B1*) and with wildtype (SL) or S5Q L7N F-ASF1B (QN) in which the amino acids (aa) at positions 5 and 7 are mutated to those in ASF1A; representative of 3 independent replicates. Note: QN F-ASF1B slightly enhances binding to H1* but not H1*B1* ST-CDAN1.
