# Supplementary material for: Mechanism of ASF1 engagement by CDAN1
Source: Nat Commun. 2025 Mar 16;16:2599. doi: 10.1038/s41467-025-57950-z (PMC11911400; doi:10.1038/s41467-025-57950-z)
Supplement: Supplementary file 2 — Description of Additional Supplementary Files [file 41467_2025_57950_MOESM2_ESM.pdf]

Description of Additional Supplementary Files

File name: Supplementary Movie 1
Description: Conformational heterogeneity of C:C:A complexes. The movie shows a progression through a cryo-EM volume series of the C:C:A complex generated through 3D variability analysis in cryoSPARC, followed by a 180º rotation, and another progression through the volume series. The same progressions are then repeated with the maps at a lower threshold to visualize low-resolution densities.
