# Supplementary material for: Mechanism of ASF1 engagement by CDAN1
Source: Nat Commun. 2025 Mar 16;16:2599. doi: 10.1038/s41467-025-57950-z (PMC11911400; doi:10.1038/s41467-025-57950-z)
Supplement: Supplementary file 4 — Reporting Summary [file 41467_2025_57950_MOESM4_ESM.pdf]

nature portfolio

Corresponding author(s): Shao

Last updated by author(s): 2/16/2025

Reporting Summary

Nature Portfolio wishes to improve the reproducibility of the work that we publish. This form provides structure for consistency and transparency in reporting. For further information on Nature Portfolio policies, see our Editorial Policies and the Editorial Policy Checklist.

Statistics

For all statistical analyses, confirm that the following items are present in the figure legend, table legend, main text, or Methods section.

n/a	Confirmed	
☐	☒	The exact sample size (*n*) for each experimental group/condition, given as a discrete number and unit of measurement
☐	☒	A statement on whether measurements were taken from distinct samples or whether the same sample was measured repeatedly
☒	☐	The statistical test(s) used AND whether they are one- or two-sided<br>*Only common tests should be described solely by name; describe more complex techniques in the Methods section.*
☒	☐	A description of all covariates tested
☒	☐	A description of any assumptions or corrections, such as tests of normality and adjustment for multiple comparisons
☒	☐	A full description of the statistical parameters including central tendency (e.g. means) or other basic estimates (e.g. regression coefficient) AND variation (e.g. standard deviation) or associated estimates of uncertainty (e.g. confidence intervals)
☒	☐	For null hypothesis testing, the test statistic (e.g. *F*, *t*, *r*) with confidence intervals, effect sizes, degrees of freedom and *P* value noted<br>*Give P values as exact values whenever suitable.*
☒	☐	For Bayesian analysis, information on the choice of priors and Markov chain Monte Carlo settings
☒	☐	For hierarchical and complex designs, identification of the appropriate level for tests and full reporting of outcomes
☒	☐	Estimates of effect sizes (e.g. Cohen's *d*, Pearson's *r*), indicating how they were calculated

Our web collection on statistics for biologists contains articles on many of the points above.

Software and code

Policy information about availability of computer code

Data collection	SerialEM v4.0.5, NIS Elements 5.21
Data analysis	Astra 7, cryoSPARC v4.3.1, ChimeraX v1.6, Coot v0.9, Phenix v1.19.2, FIJI v2.14, DeepEMhancer 20220530_cu10, Alphafold3 (alphafoldserver.com), Colabfold v1.5.5

For manuscripts utilizing custom algorithms or software that are central to the research but not yet described in published literature, software must be made available to editors and reviewers. We strongly encourage code deposition in a community repository (e.g. GitHub). See the Nature Portfolio guidelines for submitting code & software for further information.

Data

Policy information about availability of data

All manuscripts must include a data availability statement. This statement should provide the following information, where applicable:

- Accession codes, unique identifiers, or web links for publicly available datasets
- A description of any restrictions on data availability
- For clinical datasets or third party data, please ensure that the statement adheres to our policy

EM maps and models are available under accession numbers EMD-45959, EMD-45960, EMD-45961 and PDB 9CVC.

Research involving human participants, their data, or biological material

Policy information about studies with human participants or human data. See also policy information about sex, gender (identity/presentation), and sexual orientation and race, ethnicity and racism.

Reporting on sex and gender	N/A
Reporting on race, ethnicity, or other socially relevant groupings	N/A
Population characteristics	N/A
Recruitment	N/A
Ethics oversight	N/A

Note that full information on the approval of the study protocol must also be provided in the manuscript.

Field-specific reporting

Please select the one below that is the best fit for your research. If you are not sure, read the appropriate sections before making your selection.

☒ Life sciences ☐ Behavioural & social sciences ☐ Ecological, evolutionary & environmental sciences

For a reference copy of the document with all sections, see nature.com/documents/nr-reporting-summary-flat.pdf

Life sciences study design

All studies must disclose on these points even when the disclosure is negative.

Sample size	No statistical methods involving predetermined sample sizes were used. For cryo-EM datasets, sample sizes were established by semi-automated particle-picking algorithms and further classified by established cryo-EM processing pipelines.
Data exclusions	Established single particle image processing algorithms may exclude or weight particles. No other data were excluded.
Replication	At least 3 independent replicates were performed successfully for all biochemical assays.
Randomization	Established single particle image processing algorithms randomly split cryo-EM particle images into two halves during refinement.
Blinding	Blinding is not applicable to the methods reported as no group allocation was involved.

Reporting for specific materials, systems and methods

We require information from authors about some types of materials, experimental systems and methods used in many studies. Here, indicate whether each material, system or method listed is relevant to your study. If you are not sure if a list item applies to your research, read the appropriate section before selecting a response.

Materials & experimental systems

n/a	Involved in the study	
☐	☒	Antibodies
☐	☒	Eukaryotic cell lines
☒	☐	Palaeontology and archaeology
☒	☐	Animals and other organisms
☒	☐	Clinical data
☒	☐	Dual use research of concern
☒	☐	Plants

Methods

n/a	Involved in the study	
☒	☐	ChIP-seq
☒	☐	Flow cytometry
☒	☐	MRI-based neuroimaging

Antibodies

Antibodies used	Antibodies used for blotting: HRP-conjugated anti-FLAG M2 (Sigma, A8592, 1:10,000), HRP-conjugated StrepTactin (Bio-rad, 1610381, 1:5,000), HRP-conjugated anti-HA (Cell Signaling Technology, 2999S, 1:5,000), anti-CDIN1 (Abcam, ab215190, 1:1,000), anti-CDAN1 (Bethyl, A304-952A, 1:1000), anti-histone H3 (Proteintech, 17168-1-AP, 1:5000), anti-histone H4 (Proteintech, 16047-1-

	AP, 1:1000), anti-DNAJC9 (Proteintech, 25444-1-AP, 1:1000), anti-ASF1A (Cell Signaling Technology, 2990S, 1:1000), and anti-ASF1B (Proteintech, 22258-1-AP, 1:1000). Antibodies used for immunofluorescence: CDIN1 (IF: Atlas Antibodies, HPA061023, 1:50).
Validation	HRP Anti-FLAG M2 (https://www.sigmaaldrich.com/US/en/product/sigma/a8592), HRP-StrepTactin (https://www.bio-rad.com/en-us/sku/1610381-precision-protein-streptactin-hrp-conjugate-125-ul?ID=1610381), HRP anti-HA (https://www.cellsignal.com/products/antibody-conjugates/ha-tag-6e2-mouse-mab-hrp-conjugate/2999), anti-CDIN1 (ab215190, https://www.abcam.com/en-us/products/primary-antibodies/c15orf41-antibody-epr21169-28-ab215190), anti-CDAN (https://www.fortislife.com/products/primary-antibodies/rabbit-anti-codanin-1-cdan1-antibody/BETHYL-A304-952), anti-H3 (https://www.ptglab.com/products/Histone-H3-Antibody-17168-1-AP.htm), anti-H4 (https://www.ptglab.com/products/HIST1H4E-Antibody-16047-1-AP.htm), anti-DNAJC9 (https://www.ptglab.com/products/DNAJC9-Antibody-25444-1-AP.htm), anti-ASF1A (https://www.cellsignal.com/products/primary-antibodies/asf1a-c6e10-rabbit-mab/2990), and anti-ASF1B (https://www.ptglab.com/products/ASF1B-Antibody-22258-1-AP.htm) were validated by manufacturers. HRP anti-FLAG M2, HRP-StrepTactin and HRP anti-HA were also validated by over-expression of proteins tagged with specific epitopes. Anti-CDAN1 and anti-CDIN1 ((ab215190) were validated by immunoblotting of siRNA-mediated knockdown or over-expression lysates and band shift (CDIN1) or absence(CDAN1) upon tagging. We demonstrated that anti-CDIN1 (HPA061023) is not specific for IF using siCDIN-treated cells.

Eukaryotic cell lines

Policy information about cell lines and Sex and Gender in Research

Cell line source(s)	Flp-In 293 T-REx cells (R78007) and Expi293F cells (A14527) were originally obtained from ThermoFisher Scientific. CDAN-HF and CDIN-HF cell lines were generated as described in the Methods section.
Authentication	Knock-in lines were authenticated by western blotting and by Sanger sequencing of PCR amplification of the edited locus.
Mycoplasma contamination	Cells were free of mycoplasma contamination.
Commonly misidentified lines (See ICLAC register)	No commonly identified cell lines were used.

Plants

Seed stocks	N/A
Novel plant genotypes	N/A
Authentication	N/A
